# Primary care nurses’ learning styles in the light of David Kolb

**DOI:** 10.1590/0034-7167-2021-0986

**Published:** 2022-09-09

**Authors:** Led Daianna Fernandes de Figueiredo, Nair Chase da Silva, Marta Lenise do Prado

**Affiliations:** I Hospital Infantil Dr. Fajardo. Manaus, Amazonas, Brazil; II Universidade Federal do Amazonas. Manaus, Amazonas, Brazil; III Universidade Federal de Santa Catarina. Florianopólis, Santa Catarina, Brazil

**Keywords:** Nurses, Problem-Based Learning, Primary Care Nursing, Learning, Education, Continuing, Enfermeras, Aprendizaje Basado en Problemas, Atención Primaria de Salud, Aprendizaje, Educación Continua, Enfermeiras, Aprendizagem Prática, Atenção Primária à, Saúde, Aprendizagem, Educação Continuada

## Abstract

**Objectives:**

to identify primary care nurses’ learning styles in the light of David Kolb’s Experiential Learning Theory.

**Methods:**

a descriptive and exploratory qualitative study. A semi-structured interview script was used for data collection and content analysis for data processing.

**Results:**

primary care nurses showed different learning styles: diverging, which combines active experimentation and reflective observation; assimilating, which combines reflective observation and abstract conceptualization; converging, which associates abstract conceptualization and concrete experience; and accommodating, which unites concrete experience and active experimentation.

**Final Considerations:**

learning through experience requires that knowledge be understood and transformed. Nurses learn in different ways, as they have different learning styles. Therefore, recognizing nurses’ learning styles is important to foster ongoing professional development and ensure safe nursing care.

## RESUMO

**Objetivos:**

identificar o estilo de aprendizagem dos enfermeiros de atenção primária à luz da Teoria da Aprendizagem Experiencial de David Kolb.

**Métodos:**

estudo qualitativo descritivo e exploratório. Utilizou-se um roteiro de entrevista semiestruturado para a coleta dos dados, e a análise de conteúdo, para o tratamento dos dados.

**Resultados:**

enfermeiras que atuam na atenção primária apresentaram diferentes estilos de aprendizagem: divergente, que combina experiência ativa e observação reflexiva; assimilador, que combina observação reflexiva e conceitualização abstrata; convergente, que associa conceitualização abstrata e experiência concreta; e acomodador, que une a experiência concreta e a experiência ativa.

**Considerações Finais:**

aprender pela experiência requer que o conhecimento seja compreendido e transformado. Enfermeiras aprendem de modos diferentes, pois apresentam diferentes estilos de aprendizagem. Portanto, reconhecer os estilos de aprendizagem dos enfermeiros é importante para fomentar o desenvolvimento profissional contínuo e garantir um cuidado de enfermagem seguro.

## RESUMEN

**Objetivos:**

identificar el estilo de aprendizaje de los enfermeros de atención primaria a la luz de la Teoría del Aprendizaje Experiencial de David Kolb.

**Métodos:**

estudio cualitativo descriptivo y exploratorio. Se utilizó un guión de entrevista semiestructurada para la recolección de datos y análisis de contenido para el procesamiento de datos.

**Resultados:**

las enfermeras de atención primaria mostraron diferentes estilos de aprendizaje: divergente, que combina la experiencia activa y la observación reflexiva; asimilador, que combina la observación reflexiva y la conceptualización abstracta; convergente, que asocia conceptualización abstracta y experiencia concreta; y acomodante, que une experiencia concreta y experiencia activa.

**Consideraciones Finales:**

el aprendizaje a través de la experiencia requiere que el conocimiento sea comprendido y transformado. Las enfermeras aprenden de diferentes maneras, ya que tienen diferentes estilos de aprendizaje. Por lo tanto, reconocer los estilos de aprendizaje de las enfermeras es importante para fomentar el desarrollo profesional continuo y garantizar una atención de enfermería segura.

## INTRODUCTION

Nursing is a science and a profession in which its practitioners offer humanistic and scientific care. With techniques and processes for health promotion, prevention, recovery and rehabilitation, nursing offers personalized, culturally determined and safe services to individuals, families and communities^(1-4)^.

For a safe and effective nursing care, it is necessary to recognize the importance of fostering learning through experience, seeking to develop each individual’s know-how-to-be and know-how-to-do^(5)^. Continuous professional development is a strategy and an ethical commitment of nurses to promote the strengthening of the specific skills necessary for professional performance. Learning takes place throughout life, from initial training and continuously, in the context of work and in the process of continuing education. Primary care nurses recognize their training needs to improve professional performance. These include skills related to leadership and management, clinical practice, quality of care monitoring, and research practice^(6-8)^.

In his Experiential Learning Theory, Kolb argues that the individual is able to learn, create and recreate his own knowledge, based on the lived experience^(9)^. His ideas were initially thought in the field of education^(10-11)^, but have been used in different fields of vocational training^(12-14)^ as well as in the field of nursing^(15-19)^. For David Kolbe, in the learning process, practical experiences help building skills, knowledge and attitudes for problem-solving, hence the importance of fostering the acquisition of learning through experience^(9)^. It is necessary to observe how individuals learn and what is their learning preference for the construction of knowledge and what style the person adopts to learn the new.

Kolb’s Experiential Learning Theory defines that the process by which knowledge is created happens through the transformation of experience. Knowledge results from the combination of obtaining and transforming experience. For Kolb, experiential learning occurs through stages and styles. There are four stages: concrete experience (CE), in which one learns by feeling, being involved; reflective observation (RO), in which one learns by observing, reflecting, listening; abstract conceptualization (AC), which makes use of logic, reasoning, creating ideas and systematizing; active experimentation (AE), in which one learns through action, decision making, having their planned action seeking to influence the environment. The stages form a learning cycle in which knowledge will result in the union of the experience achieved and its modification^(9)^.

The combination of two stages forms a learning style. There are also four learning styles, namely: diverging, combined by CE and RO; assimilating, combined by RO and AC; converging, combined by AC and AE; and accommodating, combined by AE with CE. Learning styles are preferences of each individual in the way of perceiving, organizing, processing and understanding information. For Kolb, effective learning requires cyclical movement through the four learning styles, although there is usually preference for one style over another^(9-10)^.

In the context of primary care, nurses acquire knowledge individually and simultaneously, collectively, through the experiences lived in their daily lives, whether in the managerial scope or in their process of caring for each person or the community. In every working day, there is always something to be learned.

In order for nurses to be attentive to their continuous professional development, they should be encouraged to realize that their experiences can help them develop the necessary skills for safe care^(20)^. When perceived in the work environment and how this environment favors it, its action can be better developed. By finding their style, they can benefit from it to improve their learning. At the same time, formulators of continuing education programs can adapt their proposals to nurse characteristics. Comparing the theoretical formulations of Kolb (1984) and the experience of nurses’ daily life, we sought to answer the following question: what learning styles are manifested in primary care nurses’ daily lives?

## OBJECTIVES

To identify primary care nurses’ learning styles in the light of David Kolb’s Experiential Learning Theory.

## METHODS

### Ethical aspects

The research met the standards of Resolution 466/2012 of the Brazilian National Health Council (CNS - *Conselho Nacional de Saúde*), Ministry of Health. The researchers clarify that data collected during the research, as well as its conservation, grouping and statistical analysis, access by third parties, publication and partial or total elimination, will follow the steps established in Law 13.709/18 (General Data Protection Law). The data subjects were also guaranteed the right to secrecy and anonymization of their participation, their informative self-determination, access to the data block concerning their participation in an unrestricted manner and in an easy-to-reach form, and the unrestricted right to request a personal response in the event of publication of data that does not comply with those that have been collected. The researchers are aware that, according to Art. 42 of Law 13.709/18, due to the exercise of personal data processing activity, causing to others property, moral, individual or collective damage, in violation of personal data protection legislation, will be required to repair it.

### Study design

This is a qualitative, descriptive study developed with primary care nurses in the city of Manaus. The study followed the COnsolidated criteria for REporting Qualitative research (COREQ) norms^(21)^.

### Methodological procedure

The instrument applied is semi-structured interview type. Participants were Family Health Strategy nurses, and the technique used to carry out the research was Bardin’s content analysis for data analysis.

### Study setting and data source

The study was developed in primary care units, called Basic Family Health Units (BFHU), located in health districts of Manaus, Amazonas, Brazil, which are distributed in four districts: East, North, West and South^(22)^.

BFHU nurses who were part of the clinical nursing staff at the study site were included. Those who were on vacation or any type of leave during the data collection period were excluded. Sampling was based on the convenience criterion, and the collection outcome was based on the saturation criterion.

### Data source

Nine nurses from nine BFHU located in nine different districts of Manaus participated in the study, two in the South district, two in the West, two in the North and three in the East. The choice of BFHU was given by a prior consultation on the website of the Department of Health of Manaus. Of the participants, 6 (66.7%) were female, and 3 (33.3%) were male. Participants’ age ranged between 42 and 60 years old. The prevalent age group was from 36 to 55 years (88.9%), with a mean age of 45.7 years. Nurses’ job tenure worked at BFHU ranged from three months to 10 years, with a mean of 5.8 years, with 33.3% having worked at BFHU for up to two years, and 66.7% having worked at BFHU for more than five years.

### Data collection and organization

Data were collected between January and March 2020 through semi-structured interviews. Prior contact was made with BFHU’s managers to present the objectives and procedures and invite nurses, as well as to schedule the interview place and time.

The interview script was composed of questions to characterize the profile of participants and Kolb’s learning styles. Objective questions included full name, age, sex, time of professional activity in the strategy and time of activity in other health settings. The open-ended questions were: would you tell how was your insertion in the work process? Do you learn from your professional experience? How? Would you tell me a story of your service that generated learning? What learning opportunities do you consider to exist in the service?

The interviews were conducted by the main researcher, who conducted previous training with the researcher who supervised the study through simulation. They were performed face-to-face, audio-recorded and later transcribed manually, in full by the main researcher. They were stored in individual files, in the researchers’ personal computer, being identified by alphanumeric code: N - nurse; E - East health district, N - North, W - West; S - South. The serial number was 1 to 9. The interviews were conducted in the participants’ workplace, in an appropriate space to ensure privacy. The interviews lasted 40 minutes on average.

### Data analysis

Data analysis occurred through Bardin’s content analysis^(23)^, in three phases: pre-analysis; material exploration; and treatment of results. In the pre-analysis phase, data text skimming was performed in order to become familiar with them, analyzing and exploring the corpus of the study. In the second phase, the material was explored to find learning styles. Speeches were grouped and clipped, in order to clarify the meaning of the speeches, without the use of software. The third and last stage was interpretation of results and dialogue with existing literature on the subject.

## RESULTS

It was possible to identify the four learning styles, two nurses with diverging style (N/E-9 and N/E-8), two with assimilating style (N/W-2 and (N/N-6), three with converging style (N/E-3, N/S-5 and N/S-7) and two nurses with accommodating style (N/W-1 and N/N-4). The data are summarized in the following tables:

Chart 1 presents the stages, the diverging style characteristics and participants’ speeches that were classified in that style.

**Chart 1:** Diverging style nurses

Stages	CE and RO
Characteristics	They have the ability to look at situations from different angles, experience concrete situations and explore new opportunities.
[...]*mainly in the countryside, where there is no laboratory available 24 hours a day to perform tests and check clotting time. You take as a basis what is in literature, through books you can determine this clotting time. For this, you keep the test tube with the patient’s blood in your hand and see how long it will take to clot, this will determine how many ampoules of antivenom you will apply to the patient.* (N/E - 9) *So, well, there are things that co-workers see as difficulties, which I don’t think are, because back in the village, where I worked, there was nothing, I had to play by the ear all the time. I usually say it’s a great school, indigenous health, because you’re there in the middle of the woods, you have to think fast, you have to act, because you don’t always have communication. If something happens at night, you have to intervene without having equipment or other co-workers who can help you.* (N/E - 8)	

*CE - concrete experience; RO - reflective observation.*

Chart 2 presents the combination of stages, the characteristics present in assimilating style and the speeches of participants.

**Chart 2:** Assimilating style nurses

Stages	RO and AC
Characteristics	They experiment with new ideas, create soft technology capable of aiding care, collect and collate information using critical thinking.
*I created a spreadsheet, and, in this spreadsheet, I control the population, everyone I attend, contemplating all the programs. So, I keep track of patients with this worksheet. Here I use it, and in case anyone wants information on the total number of patients I see, I’ll find it quickly, I won’t have to look in the papers*. (N/W-2) *We have the tuberculosis program, treatment lasts for six months. There are two attack phases that are equivalent to two months and four months of maintenance* [...] *but, when it is observed that the medication is not having the desired effect, the medication is changed, and so we control through the exams every month to assess bacillary load. If they have a negative bacillary load, we have to reinforce the use of medication. And if they still have a positive bacillary charge, we have to think about what is happening, analyze patients and the environment in which they are inserted, in order to structure all the procedures and treat the patient appropriately. This, for me, is a learning experience until today, as there are changes in the protocol and in people’s lives, and we cannot let any of that go by*. (N/N- 6)	

*RO - reflective observation; AC - abstract conceptualization.*

Chart 3 presents converging style, its stages and characteristics, as well as the corresponding speeches of nurses.

**Chart 3:** Converging style nurses

Stages	AC and AE
Characteristics	They logically study ideas, conduct their assistance guided by the perception of the situation and apply their ideas in practice.
*We monitor children’s health, but sometimes the mother comes with a lot of knowledge, because she has had several children, and then she comes with their experiences, and, as much as we know that what she is doing is not it is correct, it is not correct in what we theoretically learned in college. However, with everyday life, we have to improve knowledge, we add the mother’s knowledge with ours and adapt, working with reality and making things flow, so everyone wins, especially the children, because they can eat properly*. (N/E-3) *In the home visit, we pay attention to medications, performing monitoring, because if a patient presents any changes in the exams, especially if she is diabetic, we need to intervene, or suspending the medication or decreasing the dose and always being aware of tingling, lesions, because, for these patients, lesions are more difficult to heal, it is really to draw up a map, a method to systematize care, so we can monitor them better*. (N/S-5) *We work with a group of pregnant women. These present more delicate situations and the prenatal consultation must be very thorough, as we need to be aware of the risks that this pregnant woman may present, such as hypertension in pregnancy and others, which lead to the risk of premature birth, loss of the fetus. So, we seek to see these pregnant women more carefully and, if we discover risk situations, we refer them to the doctor for assessment, to the health unit responsible for high-risk pregnancies and we have already talked about the maternity that assists this pregnant woman, so that she does not waste time looking for maternity hospitals that can assist her*. (N/S- 7)	

*AC - abstract conceptualization; AE - active experimentation.*

Finally, Chart 4 presents the combination of stages, characteristics and speeches of participants with accommodating style.

**Chart 4:** Accommodating style nurses

Stages	AE and CE
Characteristics	They execute plans, engage in new and challenging experiences.
*In my team* [strategy], *I talk a lot with them, and when there is a demand for us to do it, I read it to everyone. And if in the document they give a deadline of 30 days, I ask that we can do it in 20 days, so we can eliminate the demand and continue with our work, I don’t like to leave anything to deliver the day or two days before. Here we received the demand to re-register families and we had a deadline, we finished it before the deadline, we worked hard for it, and I always tell them that we can’t leave anything for later, if it’s to be resolved, let’s resolve it now. And I always demand goals from everyone, because it’s in the collective that you get results, you know?* (N/W-1) *If at the time I have to solve it, I get up and go look for help, I ask the patient to stay here and I go to look for it with the nurse co-worker, with the doctor co-worker of my team. If he is not, I will ask any of the other medical co-workers from another family health team, but I am not in doubt with the patient. If I have to solve it, I’ll seek help to be able to give the patient the answer, to direct her correctly, even though I don’t know now, but I leave here and I go looking for help, I go after clarification, you know? I just won’t let the patient leave without conduct, without an answer, I need to solve.* (N/N-4)	

*AE - active experimentation; CE - concrete experience.*

## DISCUSSION

Kolb considers the stages and learning styles as inseparable, i.e., to achieve the styles, it is necessary to combine the stages that can start in any of their phases, transit and combine them, thus forming a style^(9)^.

Learning through experience requires understanding and transformation to learn, absorb, resolve, and return knowledge to the environment. The way of learning is linked to actions that require the human being the ability to experience and reflect the action, creating and recreating knowledge^(16)^.

Nursing students’ experiential learning is a process of direct recognition, in which it directs students to believe, consider and execute the experiences and skills recently taught. Nursing students perform practices at the beginning of graduation. It is these practical activities that make them reflect on learning and insert what has been learned, accumulating knowledge^(24-25)^.

David Kolb addresses this grasping and understanding of learning using stages (CE, RO, AC and AE) and learning styles (diverging, assimilating, converging and accommodating), which demonstrate characteristics that are correlated with the way individuals learn. The use of development structures capable of improving situations that generate learning is linked to stages and styles. To achieve knowledge, individuals develop preferences in the way of learning, and this is marked by past experiences and current learning needs. For every two stages combined, a learning style^(9)^ is formed.

Diverging style learns best when using simulation, readings, discussions, observations. There are studies that worked with nursing students, describing that they learn from real experience and uses reflection to internalize experience^(16)^. Diverging individuals work with varied situations, seeking alternative solutions in an organized way; they relate well, are creative, recognize problems. People with diverging styles often prefer to work in groups and receive personal feedback^(26)^.

The nurses participating in this study, classified with diverging style, showed these characteristics when they established a relationship with other situations experienced. They used the analogy in the face of new situations, putting into practice an alternative solution and creativity to carry out the exam. They recognize problems and, when they observe things from another perspective, they do not see difficulties in the situations experienced and in their conduct.

Assimilating style learns using storm of ideas, questions for reflection, lectures, text reading, projects, images, diagrams and models. People with an assimilating learning style are less focused on people and more interested in abstract ideas and concepts. They prefer a concise, logical and linear approach, assimilating students as readings and having time to think about things^(26)^. The two nurses classified as assimilating individuals showed logic with the creation of a spreadsheet, moving from a way of seeing the situation to other ways of understanding that optimize the action, by gathering various information, acting systematically.

With regard to converging style, they learn better by logical reasoning, applying both theory and common sense. They are good at solving practical problems, at using hypotheses to solve a problem and make decisions, they use understanding and ideas separate from actual experience^(16,26)^. The three nurses, thus classified in the present study, presented in their speeches situations that show practical application of ideas, decision-making, resolution of technical problems and tasks.

In accommodating style, people demonstrate learning by practicing, facing challenges, taking risks. They are better suited to circumstances, prefer practical activities and experiences, and often rely on intuition rather than logic, tending to adopt a practical and experiential approach. People with this style have the ease of learning fieldwork, case studies, laboratories, simulations, homework and readings^(20,26)^. The two nurses classified with accommodating style showed that they execute plans, face the challenge, as in solving the demand before the deadline, seek to solve problems, even if they ask for help from other co-workers, because they act more by the feeling of solving a problem than by logic, as in this case to seek to help and not leave patients without meeting their demand. The nurses presented the resolution of their problems, working with other people to achieve their goals. Asking for help solving a problem with the support of others was described in another study^(27-28)^ as well as involvement in new experiences.

Learning styles combined with stages allow individuals to learn, understand and transform their way of learning through experience. Learning requires individuals to recognize the best way to gain knowledge. These ways of learning are linked to learning styles^(9)^.

Recognizing learning styles assumes relevance in the current context in which information technologies, especially the use of virtual resources, have taken a leading role in the educational context. This scenario was strongly induced by the COVID-19 pandemic experienced throughout 2020, in which remote education began to be widely used. An integrative review that synthesized evidence on podcasting in nursing and midwifery education pointed out that undergraduate nursing students who had a preference for auditory learning seemed to enjoy listening to podcasts and learned a lot from them. However, others did not like to use technology or preferred traditional teaching approaches. Also, nurses stated that they sometimes combined podcasting with other activities, such as reading lecture slides, notes, reading textbooks and discussing digital content in study groups and online forums, as this seemed to improve learning^(25,28-30)^. The findings of this study reaffirm that there are different ways to learn.

The study on students’ learning in the first period of the nursing school^(24)^ describes that, when students are led to develop practices that generate learning, they are able to transform knowledge. When teachers are encouraged to use methods capable of changing the absorption of knowledge, everyone wins, because learning from experience requires students to grasp, understand and read on the way they learn. In addition to technical skills, experiential learning can also be useful for developing nurses’ political skill and increasing interest and motivation to participate in policy formulation^(30)^.

Moreover, continuous professional development is a requirement, including to enhance the science of implementation, which plays a fundamental role in the adoption and integration of evidence-based practices to improve quality of care. Nurses need to provide safe, effective, patient-centered, timely, efficient, and equitable health care. Individuals and communities cannot wait to receive the best possible care. We now need well-equipped nurses and nursing scientists who can facilitate implementation efforts to improve health and health care^(31-32)^.

Participants’ reports about their learning experiences in different scenarios, social, cultural and technological contexts are in line with Kolb’s theory, when he considers that learning is a cumulative process of knowledge that should make sense for a learner. Nurses’ experiential learning occurs in the interaction with the environment, in the work context, being determined by the learning styles of each one. These movements make up lifelong learning, promoting continuous professional development.

Finally, there is an observation here. The study on theories by Kolb^(9)^ presented the Kolb Learning Style Inventory 4.0, which brings a new integration of learning styles into the learning cycle through the expansion of learning style types from 4 to 9 and the introduction of the concept of learning flexibility - the extent to which individuals adapt their learning style to the demands of the learning situation. The new model maintains the integration of styles across the learning cycle, emphasizing that learning requires different styles at different stages of the learning process. However, in this study, we chose to work with the four initial stages proposed by Kolb in 1984, because we understand that the model meets what we propose in this research: to highlight the importance of recognizing nurses’ different learning styles for the success of continuous professional development.

### Study limitations

The study was developed in BFHUs, and there are few studies based on Kolb’s theoretical framework about pedagogical practices in the continuing training of nursing professionals in this scenario. In this sense, this study can be replicated in other contexts of Primary Health Care, expanding the discussion of the theme and the results obtained here.

### Contributions to nursing

Being able to recognize which learning style each nurse has can facilitate the way knowledge is acquired and passed on. Therefore, nurses’ learning styles characteristics favors the process of continuing education. Acquired knowledge improvement favors the care that should be given to individuals, family and community, emerging in nursing professionals a different way of teaching.

## FINAL CONSIDERATIONS

The research data allowed us to identify primary care nurses’ learning styles in the light of David Kolb’s Experiential Learning Theory, allowing us to recognize how we can enhance nurses’ learning. Identifying, recognizing and enhancing experiential learning contributes to transforming theoretical and practical knowledge and improving the skills necessary for the performance of their duties.

The identification of different learning styles of nurses may contribute to the adequacy of continuing education programs, guiding institutional and governmental training policies. The styles demonstrate how nurses acquire learning through daily experience, which allows them to think of instructional drawings that meet the different ways of learning.

Experiential learning characteristics are the act of learning in practice, reflecting on knowledge, exploring the new, using theoretical concepts, accepting challenges. Learning styles are manifested when nurses use their practice to solve daily questions or when they think about how to solve a given situation. For these styles to be considered by managers and planners of training programs, nurses need to show how they like to learn, how they feel more comfortable seeking knowledge, whether through feelings, reflections, projections or actions.

For this, it is necessary to observe and offer tools capable of enhancing the process of learning through experience, recognizing how different learning styles can be fostered. It is necessary to understand how nurses receive theoretical knowledge and, mainly, to know what they know and how they acquire learning from practice. Thus, learning is exponentiated, improving the assistance provided to individuals, family and community by the continuing development of professional skills.
